# Mapping the sequence mutations of the 2009 H1N1 influenza A virus neuraminidase relative to drug and antibody binding sites

**DOI:** 10.1186/1745-6150-4-18

**Published:** 2009-05-20

**Authors:** Sebastian Maurer-Stroh, Jianmin Ma, Raphael Tze Chuen Lee, Fernanda L Sirota, Frank Eisenhaber

**Affiliations:** 1 Biomolecular Function Discovery Division, Bioinformatics Institute (BII), Agency for Science Technology and Research (A*STAR), 30 Biopolis Street, #07-01, Matrix, 138671, Singapore; 2 Department of Biological Sciences, National University of Singapore, 14 Science Drive 4, 117543 Singapore

## Abstract

In this work, we study the consequences of sequence variations of the "2009 H1N1" (swine or Mexican flu) influenza A virus strain neuraminidase for drug treatment and vaccination. We find that it is phylogenetically more closely related to European H1N1 swine flu and H5N1 avian flu rather than to the H1N1 counterparts in the Americas. Homology-based 3D structure modeling reveals that the novel mutations are preferentially located at the protein surface and do not interfere with the active site. The latter is the binding cavity for 3 currently used neuraminidase inhibitors: oseltamivir (Tamiflu^®^), zanamivir (Relenza^®^) and peramivir; thus, the drugs should remain effective for treatment. However, the antigenic regions of the neuraminidase relevant for vaccine development, serological typing and passive antibody treatment can differ from those of previous strains and already vary among patients.

This article was reviewed by Sandor Pongor and L. Aravind.

## Findings

The recent epidemic of the "2009 H1N1" influenza A virus (also called swine or Mexican flu) has put the world on alert since a new swine flu strain (naturally hosted by pigs) has crossed the species barrier to human and, apparently, acquired the capability for human to human transmission [1,2]. Given earlier experiences with risks of viral pandemics such as SARS and the avian flu [3], global control and public health surveillance mechanisms provided sequences of the new flu strain in public sequence databases within weeks of the outbreak. Here, we analyze the protein sequence of its neuraminidase with respect to similarities and differences to known strains and implications on drug treatment and vaccination.

### Domain architecture and posttranslational modifications

**Figure 1:**
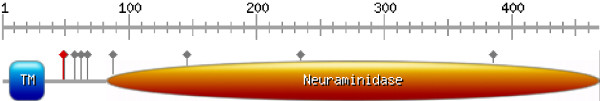
**Domain architecture (drawn with )**. Besides the labelled domains (TM ... transmembrane), grey lollipops indicate known and putative glycosylation sites and the red lollipop marks the conserved cysteine shown in Figure 2.

Sequence and residue numbering in this analysis correspond to the neuraminidase [Genbank: ACP41107.1 ] representative for the new strain. Sequence analysis was carried out following an established protocol using the ANNIE resource [4,5]. The 469 amino acid long neuraminidase (NA) protein (Figure 1) is essential for release of the viral particle from the outer membrane of infected cells by cleaving sialic acid from host glycoproteins that are recognized by the viral hemagglutinin [6]. As a type II transmembrane protein, it is N-terminally attached to the membrane [7]. It consists of a tiny cytoplasmic tail at the N-terminus (residues 1 to 6) [8] followed by the transmembrane region (residues 7 to 34) that is also responsible for translocation of the protein [9].

Next, a presumably unstructured linker region (residues 35 to 82) connects the membrane anchor to the catalytic neuraminidase domain (residues 83 to 469; Figure 1). Such unstructured linker regions are rich in small and polar residues and often harbour sites for posttranslational modifications [10,11]. Probable posttranslational modification sites in the neuraminidase of the new strain are glycosylation motifs involving N88, N146 and N235, which correspond to residues that are also glycosylated in other subtype neuraminidases [12]. However, the minimal and non-specific consensus motif of glycosylation sites (Nx [ST]) is found in total 8 times in the new strain sequence with an apparent clustering (50%) in the unstructured linker region (Figure 1). Interestingly, another putative novel glycosylation site N386, which is unique to the new strain, would be accessible on the surface, as seen in the structural models.

**Figure 2:**
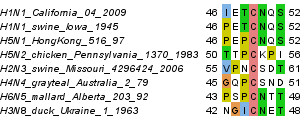
**Representative alignment of the sequence environment of the conserved cysteine C49 that could either serve for intermolecular disulfide bridges or as palmitoylation site**.

Comparing among all strains, the sequence variation is largest in the linker region, including large deleted segments. Nevertheless, this region harbours a cysteine (Figure 2) that can be aligned over multiple NA subtypes and is conserved in N1-N5 and N8, but not in N6, N7 and N9. Earlier reports assume that, at least in related viruses, cysteines in the non-globular region could be involved in intermolecular disulfide bridges [13-15]. Alternatively, by analogy to other influenza proteins such as hemagglutinin [16] and M2 protein [17], it cannot yet be excluded that cysteine C49 is palmitoylated and that the anchor localizes the protein to lipid rafts [18].

### Phylogenetic relation of new NA to known subtypes

Influenza A virus protein sequences were downloaded from NCBI (as of April 29^th^). Neuraminidases were identified by BLAST (E-value < 0.001) [19] using the representative NA of the new strain as query [Genbank: ACP41107.1 ]. Redundancy was removed with cd-hit at a level of maximal 90% sequence identity [20], the remaining sequences were aligned with MAFFT (using L-INS-I settings [21]) and the resulting multiple alignment was visualized and annotated in Jalview [22]. A neighbour joining tree with pairwise gap deletion, Poisson correction as distance measure and 500 bootstrap replicates (generated with MEGA [23]) produces robust groupings consistent with previous studies [24] for the known NA subtypes (clustering of N1, N4, N5+N8 on one side and N2, N3, N6+N7+N9 on the other) and reliably places the new NA with other N1s. Interestingly, inside the N1 cluster, the new NA appeared close to the N1 of H5N1 avian flu viruses. The alignment and corresponding phylogenetic tree are available at .

**Figure 3:**
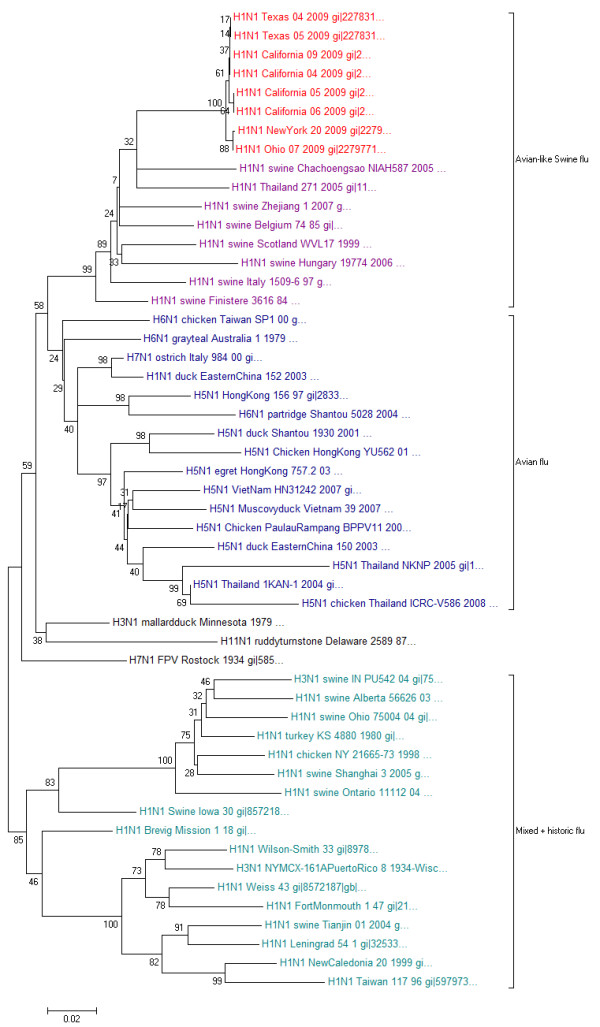
**Phylogenetic tree of neuraminidase protein sequences of the N1 subtype family**.

Hence, we repeated the analysis (same protocol as outlined above) for a detailed mapping of only the N1 subtype family with the difference of allowing 95% sequence identity for sequences before 2009 but keeping all new NA sequences (as of April 29^th^). A characteristic clustering emerges (Figure 3) that roughly corresponds to host and geographic distributions, consistent with previous reports [25,26]. The observed clustering is robust in respect to the method used for tree generation (same for maximum parsimony or neighbour joining trees with JTT distance and gamma-distributed variable rates). The 2009 NA is part of a cluster of avian-like swine flu H1N1 strains predominantly found in European pigs. However, previous examples of human infections from swine flu are also part of the same cluster, for example from 2005 in Thailand [27]. This indicates that, similar to the current outbreak, closely related H1N1 strains have crossed species boundaries on previous occasions as also evidenced by further reports in the literature [28-30].

Moreover, neuroaminidases of these new H1N1 swine flu examples are more similar to H5N1 avian flu strains than other H1N1 variants found in the Americas or than that of the historic strains such as the 1918 Spanish flu [31]. This is surprising since avian flu strains typically have different hemaglutinin (HA) subtypes (e.g. H5N1). Combinations of HA and NA subtypes need to be fine-tuned to recognize the same type of sialic acid modifications to allow smooth interplay of the two proteins, which is important for the viral cycle [6]. These results support the notion that, also inside the family of N1 subtypes, a clear distinction can be made between avian-like H1N1, such as the one from the current outbreak, and other existing H1N1 strains.

### Structural modelling and mapping of new mutations

The crystal structures of both the historic 1918 NA as well as the avian flu NA are available in complex with currently used drugs. We created a homology model of the new 2009 swine flu NA to map the sequence differences to the three-dimensional structure templates. Using Modeller [32], the sequence of the new neuraminidase [Genbank Accession: ACP41107.1 ] was modelled 50 times onto multiple templates (PDB: 2hu4[33], 3ckz[34], 3b7e[35], 3beq[35]) and the resulting best model (as judged by DOPE score) further refined with short simulated annealing MD simulations in the presence of a bound inhibitor (zanamivir, oseltamivir or peramivir) as implemented in the Yasara Structure package [36]. The final atom-resolution models are available in PDB format at .

**Figure 4:**
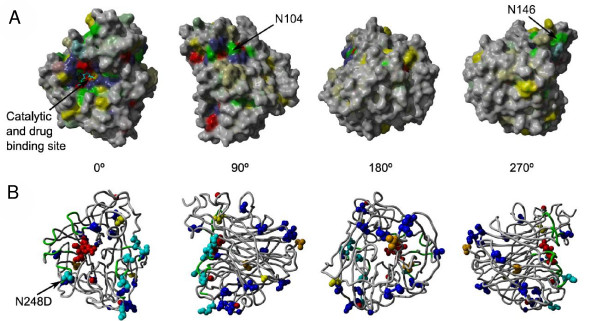
**A) Surface representation of the structural model of the neuraminidase domain of the new strain in complex with zanamivir**. Coloring is based on sequence conservation over all NA subtypes. Grey means no conservation. Other colors are according to physical properties: yellow ... hydrophobic, green ... polar, blue ... positive charge, red ... negative charge. Color intensities are proportional to strength of conservation. **B) Mapping of new mutations to structure**. Cyan colored residues are mutations at typical antibody recognition sites. Blue residues indicate differences to both the H5N1 avian flu as well as H1N1 from the 1918 Spanish flu. Yellow residues are intra-strain variations occurring in multiple patients of the 2009 H1N1 outbreak and orange if they have only been found in isolated patients, so far. Note that the intra-strain variation N248D is colored cyan since it is part of the antibody recognition site. The backbone of the antibody recognition sites is colored green and the bound drug and 3 calcium ions are shown in red.

We mapped the level of residue conservation (calculated with the evolutionary trace algorithm [37]) from the multiple alignment of all NA subtypes to its corresponding position in the structure. The results show the strict conservation close to the neuraminidase catalytic site, which also serves as the drug binding pocket (Figure 4A). The remaining conserved patches (for example the sites around N104 or below N146) fit into each other and form the dimerization/tetramerization interfaces [35]. A model of the dimeric version is available in PDB format at .

**Figure 5:**
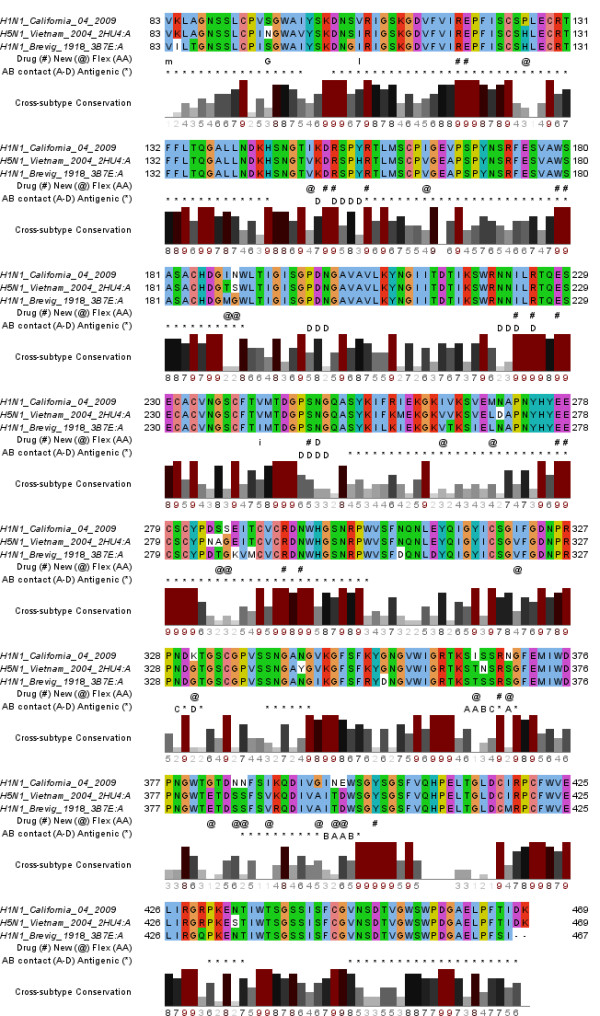
**Alignment of the NA domain of the 2009 H1N1 strain with the sequences in crystal structures of H5N1 avian flu as well as H1N1 from the 1918 Spanish flu**. Residues within 3 Å of the bound drug are indicated with "#", while residues that are different in the new strain compared to both other structures are marked with "@".Intra-strain variation (Flex [AA] in the first annotation line) is displayed as the respective mutated residue in capital letters if found in multiple patients (e.g. D for the N248D substitution) or lower-case (e.g. "i" for V241I) for single occurrences. In the second annotation row, antigenic regions are labelled as "*". Residues with < 3 Å contact to antibodies are labelled "A" for interactions derived from PDB:1ncb, "B" from both PDB:1ncb and PDB:1nmb, "C" from PDB:1nmb and "D" from PDB:2aep.

Next, we compared the sequences of the new strain with the related H5N1 from avian flu and H1N1 from the Spanish flu (Figure 5). Among 387 residues that were structurally modelled, the "2009 H1N1" neuraminidase differs from the other two in 21 positions. The mapping to the structure (Figure 4B) shows that the novel sequence mutations are distributed all around the surface of the molecule leaving the hydrophobic core, but also the catalytic site, essentially untouched. Importantly, none of the new mutations appears sufficiently close to affect the drug binding pocket. For example, all 17 residues within 3 Å of the zanamivir molecule bound to the active site are fully conserved among all three strains. The closest mutation is the conservative V149I substitution at a distance of ~10 Å to zanamivir and ~7 Å to oseltavimir.

It has to be noted that indirect effects of the mutations that may alter the binding pocket also from a greater distance are difficult to assess and cannot be excluded. To this extent, we have analysed coevolution patterns in an extensive alignment of more than 6000 non-identical Influenza A neuraminidases to eventually identify connected networks of residues using the SCA algorithm [38] as implemented in [39], but no network that would connect the surface directly to the core and catalytic site was found (see supplementary material). In fact, all positions of the observed mutations are at the surface and naturally variable, as judged by the conservation and SCA analysis, which would rather indicate that they do not have an effect on the structure of the more distant binding pocket.

Thus, we conclude that the drug binding pocket remains unchanged in the new strain and, hence, the binding behaviour of neuraminidase inhibitors such as oseltamivir (Tamiflu^®^) and zanamivir (Relenza^®^) should be unaffected. Indeed, initial clinical reports suggest that the new virus is susceptible to the two drugs [40]. Our findings support this notion and provide a molecular mechanism. Furthermore, the third currently tested neuraminidase inhibitor, peramivir, should also be effective since it also shares the same binding pocket.

Next, we review how the new mutations affect vaccine development through altering antibody interactions as well as antigenic regions. There are 3 crystal structures of related neuraminidases in complex with antibodies [41-43]. In Figure 5, we annotate residues that are within 3 Å distance to the respective bound antibody and, hence, crucial for the interaction. Interestingly, residues in sites recognized by both NC41 and NC10 antibodies appear mutated in the new strain. This would suggest that these old antibodies (that were originally directed against N9 neuraminidase) would probably not bind to NA of the new strain. Nevertheless, using the same regions as epitope may be a viable option for novel vaccine development.

Additionally, several other known antigenic regions, partially derived from surviving patients of previous flu outbreaks (e.g. H5N1), are reported in the literature [44-46] and their location is indicated in the alignment (Figure 5). Another extensive source of epitopes and antigens including neuraminidase of influenza A viruses is the immune epitope database (IEDB) [47] and the complete mapping of epitopes for the new H1N1 NA sequence is available at . While several of the new mutations are found in antigenic regions, it is also apparent that they often occur on positions that are hardly conserved among different NA subtypes. Consequently, these regions are evolutionarily more flexible and may mutate fast. This increases the risk of evading antibody responses of human hosts acquired during previous flu infection or from vaccination.

### Sequence variation among patients with the same 2009 H1N1 strain

After the first wave of new patient sequences arrived, it becomes clear that there are at least two major lineages that are distinguishable by only few mutations. Most notably, N248 has mutated to Aspartate (D248) in the New York infection cluster. All intra-strain mutations available before May 8^th ^2009 are indicated in Figures 4B and 5. As expected, they are predominantly found on the surface and in regions that are known to be variable from the conservation analysis. While the drug binding pocket remains unaffected by these most recent mutations, the N248D substitution changes a central part of an antibody recognition site. This has important consequences for vaccine development, forcing to either avoid this epitope or produce combined vaccines to account for the epitope variation observed in different patient groups. Although the mutation pattern will become less transparent over time it may still serve to delineate chains of transmission, retrospectively.

## Conclusion

In summary, we provide a sequence analysis and structural modelling of the neuraminidase from the 2009 H1N1 swine flu outbreak. Besides mapping of phylogenetic relationships to other strains, we find that the sequence variation in the new strain does not seem to affect the drug binding site but may very well alter common epitopes. To allow quick analysis of future mutations that could produce drug or vaccine-resistant strains, we provide a tool for 3D visualization of the neuraminidase structure models with mapping of drug and antibody recognition sites on the supplementary webpage .

## Abbreviations

NA: neuraminidase; HA: hemagglutinin.

## Competing interests

The authors declare that they have no competing interests.

## Authors' contributions

SMS did the alignments and phylogenetic trees. FLS contributed the domain architecture analysis and helped with the phylogenetic analysis. MJ did the structural models and conservation mapping. RLTC contributed the study on antigenic regions and the Jmol visualization on the webpage. SMS and FE wrote the manuscript; all authors approved the final version.

## Reviewers' comments

### Reviewer 1

Sandor Pongor, International Centre for Genetic Engineering and Biotechnology, Trieste, Italy

*In this work, the authors carry out a predictive analysis of the "2009 H1N1" (swine or Mexican flu) influenza A virus strain, based on phylogenetic analysis and 3D homology modelling. The results show that this strain is phylogenetically more closely related to European H1N1 swine flu and H5N1 avian flu rather than to the H1N1 counterparts in the Americas*.

*Homology-modeling of the neuraminidase reveals that the novel mutations are not likely to interfere with the active site so the currently used neuraminidase inhibitors (oseltamivir, zanamivir and peramivir) will be effective against the new virus strain*.

*The subject is very timely and the approach is adequate. The authors may want to include analysis of more patient data that were published since the analysis was completed. More and more sequences are being published from all over the world that might be worthwhile to include into this analysis. The authors may consider establishing a periodically updated homepage, if appropriate. In summary, the analysis is careful and carried out in a commendable fashion, and the findings are highly significant*.

#### Response

Indeed, there have been additional "2009 H1N1" neuraminidase sequences since the submission of this manuscript for review. Between April 29^th ^and May 8^th^, 45 new sequences became available. Overall, 3 mutations occur in multiple (S95G, V106I and N248D) while 2 mutations are restricted to single patient virus isolates (V83M, V241I), so far. We have included a section about these mutations identified in new patient sequences and mapped them to the structure. As discussed in the main text, the intra-strain variation is typically found on the surface and at positions expected to be variable as judged by the conservation analysis among all NA subtypes. Interestingly, one of the mutations among patients (N248D) is critically affecting one of the antibody binding sites.

As the virus will continue to evolve, new mutations will become available and the best way we have found to allow quick mapping and update of new sequence variation in respect to drug and antibody binding sites is to give full access to users/readers via a 3D structure visualization tool at the supplementary webpage  (instructions to map new mutations are given, including an example).

### Reviewer 2

L. Aravind, National Center for Biotechnology Information, Bethesda, MD, USA

*Maurer-Stroh et al discuss sequence features of the neuraminidase gene of the virus behind the latest influenza outbreak, 2009 H1N1. Of the features of interest they note that this strain has acquired a novel glycosylation consensus site that could be surface accessible. Of course, it remains unknown if this site is indeed used for modification. The key finding in the paper is that the binding cavity for neuraminidase inhibitors is unaffected as suggested by molecular modeling. This finding is of significance in the current situation of an outbreak with pandemic potential. However, one issue needs to be highlighted in this regard – mutations far away from the binding site can potentially affect the shape and or binding affinity of the binding pocket. These are not always captured by homology models, especially the issue of affinity. In principle, authors could use a conservation patterns or co-evolution measures (e.g. as in PMID: 10514373) to determine if there are interaction chains that might connect distant residues to the active site. In the least it would be useful to provide the caveat of distant changes affecting affinity in the current paper*.

#### Response

We totally agree with the referee that also mutations at a greater distance may affect the binding pocket under special circumstances and we have added a new paragraph to the manuscript. However, we have to admit that it remains essentially impossible to quantify the influence of non-direct interactions by theoretical means unambiguously. In our experience, co-evolution measures such as the one proposed and several others (PMID: 18056067) produce high rates of false positives and, therefore, the interpretation is difficult. We did the requested analysis with Ranganathan's SCA algorithm to eventually identify connected networks of residues using the webserver implementation at the Gerstein lab, but no network that would connect the surface directly to the core and catalytic site was found (see supplementary webpage for full details). In fact, all positions of the observed mutations are at the surface and naturally variable, as judged by the conservation and SCA analysis, which would rather indicate that they do not have an effect on the structure of the more distant binding pocket. A possible problem why the SCA could not work in this case is the high level of sequence similarity among the neuraminidases which only gives limited numbers of informative correlated mutations. This is a totally different scenario from alignments of highly divergent sequences that still have the same fold, such as the small PDZ domains analyzed by Ranganathan. In the case of a diverse family with shared fold, correlated mutations are indicative of allowed fluctuations also among structurally important residues. However, with the neuraminidases, most variation can be attributed to surface residues, which makes sense given the pressure to avoid immune responses. Additionally, sequence sampling of neuraminidases is not independent but biased by transmission chains and clusters of outbreaks. A possible but also not necessarily more precise alternative to judge indirect effects on drug binding would be to run free energy simulations with the bound drug to judge changes of affinity caused by the mutations but this is a tricky and time-consuming endeavour that would burst the scope of this current manuscript.

unspecific consensus motif: change to "non-specific"

#### Response

changed.

*Phylogenetic analysis: While for sequences at this range of similarity Poisson correction may not have negative consequences it is definitely better to repeat the analysis with JTT and variable rates to see if the clustering remains the same or changes drastically*.

#### Response

We confirmed that the tree clustering inside the N1 subtype family is robust regarding different tree generation methods and added this also to the text. We also provide the suggested JTT distance tree with variable rates as supplementary at .

*" This indicates that, similar to the current outbreak, scenarios of breaches in the species barrier between human and pigs have already arisen out of closely related H1N1 strains as also evidenced by further reports in the literature *[26,27].*" The wording of this sentence is somewhat unclear. It appears that the authors wish to state that H1N1 like strains have crossed species boundaries on other occasions, but this is not necessarily clear in the sentence*.

#### Response

changed to "This indicates that, similar to the current outbreak, closely related H1N1 strains have crossed species boundaries on previous occasions as also evidenced by further reports in the literature".

*"These results support the notion that, also inside the family of N1 subtypes, a clear distinction can be made to distinguish avian-like H1N1, such as the one from the current outbreak, from other existing H1N1 strains." What would be the explanation for this? A recent recombination between an avian-like H1N1 or has it diverged from other avian like H1N1 with the recombination occurring much earlier. Could this information be superimposed in phylogenetic context on current figure *3?

#### Response

This is, of course, a very interesting question and difficult to deduce from the phylogenetic tree of a single protein without molecular clock, as in our case. However, this and similar questions have already been analyzed to quite some detail for the existing strains. The current knowledge of the scenario for H1N1 is that around the late 1970s to early 1980s, a human-avian reassortant virus started to be detected in European pigs as host (PMID: 8091678). Then, this avian-like swine flu started to move from the European continent to the UK in the early 90s (PMID: 9049404). More reassortments and emergence of the N2 subtype that quickly spread is also well documented. This phylogenetic analysis plugs the new H1N1 strain into the already known clusterings among the NA subtypes and within the N1 family.
